# A Regional Categorization for “New-Type Urbanization” in China

**DOI:** 10.1371/journal.pone.0134253

**Published:** 2015-08-03

**Authors:** Chuanglin Fang, Haitao Ma, Jing Wang

**Affiliations:** 1 Institute of Geographic Sciences and Natural Resources Research, Chinese Academy of Sciences, Beijing, China; 2 Key Laboratory of Regional Sustainable Development Modeling, Chinese Academy of Sciences, Beijing, China; 3 Beijing Academy of Social Sciences, Beijing, China; East China University of Science and Technology, CHINA

## Abstract

Regional differences in the character of urbanization in China are substantial. The promotion of what has been termed “new-type urbanization” cannot, as a result of these regional differences, be expected to follow a universal approach—rather, such a development must objectively adhere to locational and category-specific principles and adopt differentiated urbanization development models. Regional categorization is often used in geography, but is rarely deployed in research addressing human and social problems relating to urbanization. In March 2014, China published the *National New-type Urbanization Plan (2014–2020)*, which calls for the scientific and reasonable planning of “new-type urbanization,” and appropriate regional categorizations are urgently needed in order to guide this reform. Responding to this challenge, this research engaged in the design of a “dominantly quantitative analysis, qualitatively supplemented” method in order to divide China into 5 main regions and 47 sub-regions in terms of new-type urbanization. The paper discusses the features and key problems of each region. This study introduces a new method for regional categorization, thereby remedying the lack of regional categorization in relation to “new-type urbanization” in China, and ultimately promoting the development of regional categorization in the humanities as a valuable reference for healthy and sustainable Chinese urbanization.

## Introduction

China is presently undergoing rapid urbanization; in the past two decades, the country’s urbanization has attracted widespread international attention from politicians, academics, and business circles [1–6]. Nobel Laureate Joseph Stiglitz has in fact described China’s urbanization as one of two “keys” to mankind’s development in the twenty-first century (the other being the USA’s technological innovation) [7]. In 2002, the Chinese government for the first time explicitly expressed their desire intent to “take the first step on the road to an ‘urbanization with Chinese characteristics’,” an important recognition of China’s transformation from traditional patterns of urbanization to what in Chinese policy terms is referred to as “new-type urbanization.” To facilitate the healthy development of China’s cities, the Chinese Government issued the first outline of their urbanization plan in March 2014: the *National New-Type Urbanization Plan (2014–2020)* sets out a blueprint for China’s future urbanization and economic development. In accordance with that plan, “new-type urbanization” will thus form an important model in relation to China’s urban construction, social development, and economic growth in coming decades [1]. China is an exceedingly large country, with great regional differences in the rate and character of its urbanization—as such, the promotion of new-type urbanization will not be uniform across the country’s regions but rather will (and indeed must) vary [8]. At present, China has not yet categorized different types of urbanization regions or established specific guidelines for such a procedure [9]. For this reason, research addressing new-type urbanization and its regionalization has become essential. Such research offers the possibility of underpinning regionally specific discussions of the objective, focus, direction, and approach of new-type urbanization, and thereby of facilitating such urbanization in a manner that is responsive to the diverse conditions of China’s regions.

### Literature Review and Theoretical Framework

The “new-type” urbanization model represents a strong departure from the traditional processes of urbanization in China, wherein urbanization patterns underwent an evolution from economy-centered to human-centered patterns (a shift that occurred in step with the economic and social development of the nation) [1]. Compared to this “traditional” urbanization process, new-type urbanization places more emphasis on the universal coverage of social and public services, the service economy, the integration of local culture and urban development, ecological and environmental protection, and innovation in urban and rural management. As Chinese Premier Li Keqiang puts it, new-type urbanization should be “people-oriented”—as such, any behavior or activity related to the promotion of urbanization via construction must consider human factors from the outset, ultimately aiming to bring about “the comprehensive development of man” [10]. The concept of new-type urbanization is developed in an iterative manner within related policy, with the highest guideline being the delivery of “intensive and efficient, people-oriented, ecologically livable, fair and just, inclusive and harmonious” environments. Since the Chinese Government regards new-type urbanization as an engine for future development and for the expansion of domestic demand, the model has garnered wide attention and aroused lively discussion from almost every circle within Chinese society [11].

Discussions of new-type urbanization have primarily revolved around themes of population agglomeration, land use, income increase, and the equalization of public services and cultural heritage. Firstly, “urbanization” generally refers to the urbanization of a population, and as such it must be recognized that in the course of population migration, many new immigrant send up working in cities without the treatment or welfare due to them as citizens of those cities, a situation which brings to the foreground a range of social fairness issues [12]. Further, new urban populations arrive from both from the rural hinterlands of existing cities and from more remote locations, and this complicates the coordination of urban-rural development, and of inter-city development [13]. In addition, the excessive concentration of population in large cities results in the emission of pollutants, causing serious urban environmental problems. For example, although China’s urban agglomerations produce more than three quarters of the country’s total economic output, they also produce more than three quarters of the country’s total pollution; this outcome is vividly reflected in the large-scale haze of pollution that has settled over the urban agglomerations on the east coast and in the northeastern region [14]. Secondly, land urbanization is also the focus of a number of scholarly discussions [2, 15–17]. Foreign research on urbanization considers arable land to be positively correlated with urbanization, suggesting that the faster the rate of urbanization, the more arable land there will be [18]. However, in China, rapid urbanization has lead to a drastic decline in the country’s cultivated land area [19]. At present, China’s urban land-use growth elasticity coefficient (urban land growth rate/ urban population growth rate) fluctuates between 1.36 and 2.30, which is much higher than the world standard in terms of a reasonable limit of 1.12 [20]. To address this problem, a debate on the abolition of China’s 1.8 billion mu arable land red line has been launched by the country’s academic community. Some scholars partaking in this debate have suggested that the arable land red line should not be abolished and that land-use efficiency must be improved; others have argued that the arable land red line can be exceeded and that the key issue is rather increasing food production [21]. Some have alternately proposed that a balance can be achieved by methods of supplementing urban construction land and rural farmland [22]. The third key issue addressed by existing literature in relation to new-type urbanization is its potential to increase levels of domestic consumption on the basis of increased income [23]. Various scholars have discussed the problems with and possible paths towards increasing revenue in agricultural areas, forest and pastoral areas, mountainous areas and coal zones under different natural resource conditions [24]. This previous research lays a solid foundation for the option of adjusting measures to local conditions. Fourthly, the equalization of public service—meaning that everyone in a city should be able to access and enjoy that city’s public services—is one of the ideal goals of new-type urbanization. Within this category of comment, the particular standard to which such services must be held is heavily disputed: should it be the national minimum standard, or the average level, or the equality of opportunity? No unified position or understanding has been reached in relation to this divisive point [25]. Lastly, cultural development consistently returns as a key topic, with the future development of new-type urbanization hanging partly on its ability to face up to cultural demands [26]. In the current large-scale urban development which is seen across China, many of the traditional cultures, national cultures, and religious cultures are not protected and thus not passed on, leading to a lack of urban culture in the era of globalization, a situation wherein cities lose their “light” by losing their cultural richness. It can be seen from the various discussions that are summarized above that China is feeling its way forward in relation to “new-type” urbanization; whilst many issues will never be able to be resolved in a conclusive manner, they highlight a row of more specific problems which require equally specific solutions.

As a big country, China displays significant regional differences: different regions present their own distinct sustainable development problems in relation to “new-type” urbanization. In turn, these challenges necessitate a range of different kinds of research to be undertaken [27], including: research into informal institutions in densely populated areas [28], research into biological communities in Southwest China [29], research into land reclamation of Northeast China [30], and research into the different types of rapid urbanization in eastern coastal areas [9]. There is also a lot of comparative research being undertaken into the urbanization of various areas [31]. With a view to such comparisons, new-type urbanization can be expected to perform quite differently in each of China’s regions: areas each faces different key problems and necessitates consideration of different issues. An insistence on the recognition of this regional specificity forms the basis of this study, the goal of which is to divide the country into a number of “new-type urbanization regions,” in order to facilitate the solution of regionally specific problems. Resolving regional disparities through regional division is in fact a common method in geography [32], which is now not only used to address questions of natural geography but also to solve problems within economic geography and the humanities [33]. In order to fully encompass the scope and possibilities of the humanities in addressing social problems, any regional categorization must make good use of existing methods. This is true of the categorization undertaken in this study, which specifically addresses China’s “new-type” urbanization and its regional implications.

An analysis of the existing literature on new-type urbanization in China can be used to construct a theoretical framework for the regional categorization of new-type urbanization. The essence of new-type urbanization lies in promoting the development of a series of key elements, namely: population, land, income, public services, and culture in the urbanization process [34], subsumed beneath the ultimate goal of achieving “people-oriented urbanization.” These five key elements themselves have a range of different emphases. The first of these policy directives, “the healthy development of the population,” focuses on addressing the unfairness and inequality experienced by agricultural immigrants, as well as the diseases resulting from excessive population agglomeration, and the lack of coordination between urban and rural development and between cities. These directives therefore primarily concentrate on China’s densely populated urban agglomeration areas [4]. The second bracket of directives, which address “the healthy development of the land,” focus on the significant reduction of arable land, the increasing food gap, and urban and rural land replacement [35]; these tend to relate most strongly to the major grain-producing areas. The third set of directives, related to “the healthy development of income,” focus on achieving steady and sustainable steady increases in income through the characterization and specialization of industrial development [36]. These can be associated with areas linked to the agricultural, forestry, and animal husbandry industries (taking into account local characteristics of these industries in the Chinese context). The fourth element, which is most relevant to the contiguous poverty-stricken areas, addresses “the healthy development of the public service” and focuses on promoting the equalization of public services, social fairness, and justice. Finally, “the healthy development of the culture” focuses on the convergence of urban construction, national unity, and religious issues, and is targeted at the ethnic autonomous areas. Despite their diverse points of departure, these five elements are correlated: population is the core issue, land is the supporting power, income is the impetus, public services are the guarantee, and culture is the heritage. Together, they facilitate new-type urbanization. With the aim of adjusting these measures to meet local conditions [37], and through reference to a variety of regional categorization methods, this study matches the five key elements and their corresponding key issues to different regions with distinct characteristics, thereby categorizing Chinese new-type urbanizationwith respect to five main regions. We then go on to discuss the main problems and their solutions via a consideration of the features of each area. This categorization is performed with the aim of promoting healthy and sustainable urbanization in China [38].

## Materials and Methods

### Materials

This study primarily addresses two types of materials. The first type comprises indicators for cluster analysis, taken primarily from the *China Statistic Yearbook (2013)*. The entire study used data from 2012. The other type of material relates to national planning and division maps, primarily sourced from government websites, including the following: the *National New-Type Urbanization Plan*; *National Major Function-Oriented Zone Planning*; the *Medium and Long-Term Plan for National Food Security (2008–2020)*; the *Outline for Development-Oriented Poverty Reduction for China's Rural Areas (2011–2020)*; the Urban Agglomeration Development Plan of China; *China’s Comprehensive Agricultural Regionalization*; and *China’s Ecological Function Regionalization*.

### Methods

This study first sets out a series of principles for new-type urbanization planning. These are: (1) Comprehensiveness, which refers to comprehensively considering population allocation, urbanization level, social and economic development conditions, natural conditions within the region, and the nature and development direction of cities in various types of urbanization development regions; (2) Dominance, which refers to finding one or more dominant elements that lead to various division characteristics and using those elements as the basis for characterization of new-type urbanization regional divisions; (3) Consistency, which refers to paying attention to the consistency of dominant element features in categorizing regional units of new-type urbanization—for instance, the consistency of the substance of urbanization features, environment of regional development, and direction of development, as well as general similarity of the urbanization development level; (4) Regional Features, in that the smallest regionalization units should be continuous regional units; and (5) Feasibility, which refers to consideration of the feasibility of implementing regionalization. The basic unit of regionalization in China’s administrative division is the county. When the dividing lines of various types of regions are complex and regional features are not distinct, administrative divisions one level up can be properly considered.

**Fig 1:**
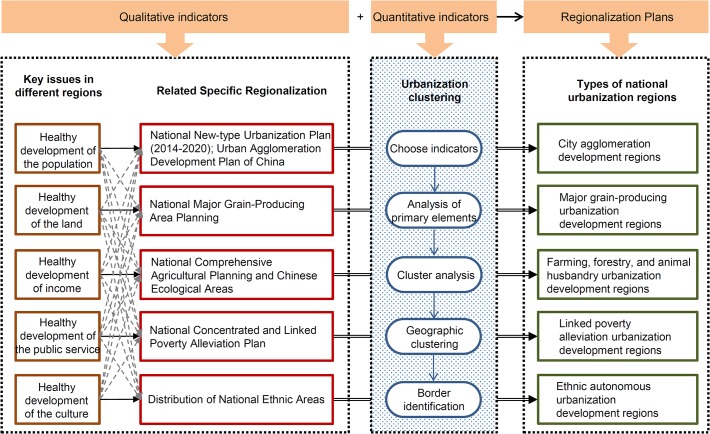
Method and ideas for designing a Regional Categorization of New-Type Urbanization in China.

Based on the principles elucidated above, as well as the features of new-type urbanization and the objectives of categorized and regionalized management, this study utilizes a “dominantly quantitative analysis dominated, qualitatively supplemented” method, which we designed for in order to produce a proposal for the comprehensive regionalization of new-type urbanization in China (Fig 1). This method integrates qualitative and quantitative indicators, using qualitative indicators as the primary basis for division and quantitative indicators as the primary basis for setting borders. Based on the interacting effects of the two types of indicators, this study completes a regionalization of new-type urbanization in China.

Qualitative indicators constituted the primary basis for the division of the country into regions in the study, allowing us to address two problems: how many regions there should be after division, and how should these regions be divided? In accordance with the core ideas of new-type urbanization as expressed in relevant policy guidelines, and with a view to the five key elements presented in the theoretical framework, we propose five main regions, which we see as being appropriate in meeting the goal of facilitating the healthy development of different regions through new-type urbanization processes, as well as in addressing the unique problems faced by areas across the country. The category “urban agglomeration areas” is formulated in order to address population urbanization issues in the densely populated urban agglomeration areas, and based mainly on the *National New-type Urbanization Plan (2014–2020)* and the *Urban Agglomeration Development Plan of China* (S1 Fig) [14, 39]. The category “major grain-producing areas” addresses the land urbanization issue in relation to rapidly decreasing arable land areas, and is formulated mainly on the basis of the *National Major Grain-Producing Area Planning* (S2 Fig) (http://www.china.com.cn). We have included “farming, forestry, and animal husbandry areas” as a third category in order to deal with the special industries and income increases, defining this category mainly via data sourced from *National Comprehensive Agricultural Planning* (S3 Fig) (http://nc.mofcom.gov.cn) and *Chinese Ecological Areas* (S4 Fig) (http://www.zhb.gov.cn). “Contiguous poverty-stricken areas” constitutes a category in the study introduced in order to address the equality of public services; the definition of this category was based mainly on data from the *National Concentrated and Linked Poverty Alleviation Plan* (S5 Fig) (http://www.fcpmc.org). Based mainly on the *Distribution of National Ethnic Areas* (S6 Fig) (http://www.mofangge.com), “ethnic autonomous regions” constitutes a fifth category, which is used to deal with the local characteristics of cultural heritage. In the situation where an area was found to belong to multiple types of regions, its category was determined according to the following hierarchy: city agglomeration areas was prioritized first, followed by major grain-producing areas, farming, forestry, and animal husbandry areas, linked poverty alleviation areas, and finally ethnic autonomous regions.

Quantitative indicators constituted the basic references for establishing borders in the study. The analysis of such indicators was undertaken in five steps. The first step was to choose the indicators; we chose multiple indicators to satisfy the “comprehensiveness” principle of regionalization. The second and third steps comprised the analysis of primary elements and cluster analysis. We used SPSS 19.0 software to conduct these analyses, which reflect the “dominance” and “consistency” principles. The fourth step was geographic clustering. We referred to the ArcGIS 10.1 software platform in order to conduct the analysis, which reflected the “regional feature” principle. The fifth step was border identification; here, we conducted an analysis according to the dominant features of various regions and administrative borders, in a manner which reflected the “feasibility” principle.

The method proposed here differs from that of existing studies in a number of important ways. Since the new-type urbanization model emphasizes the humanities via its focus on social problems, our method was mainly qualitative, treating quantitative indicators as supplementary (although previous natural zoning and economic divisions were established quantitatively). This approach is closely related to the research objective of providing new ideas and methods for studying the categorization of human issues.

## Results

### Urbanization Clustering

**Fig 2:**
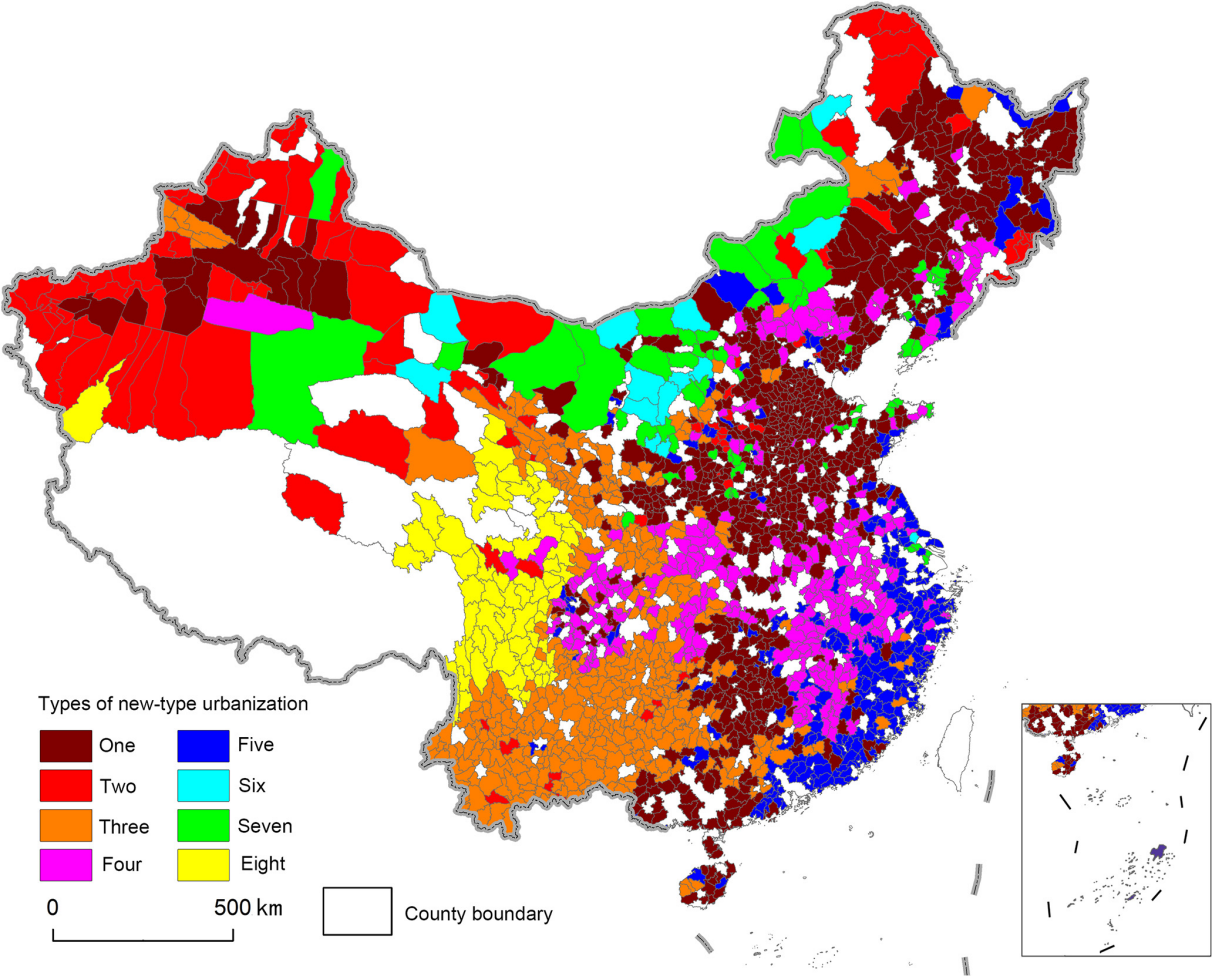
Cluster Analysis Diagram of Comprehensive Regionalization in China’s New-Type Urbanization.

Following an analysis of the factors influencing the development of urbanization processes in China, the study selected 13 indicators for analysis, including: gross domestic product (GDP) per capita, fixed assets investment per capita, the percentage of employees in manufacturing industries, the percentage of employees in producer service industries, the percentage of employees in consumer service industries, average years of education, the percentage of professional technicians, fiscal income per capita, the percentage of migrating population, the number of medical beds per 10,000 people, the number of welfare house beds per 10,000 people, distance to railroads, topographic relief amplitude, and water abundance. By using SPSS to undertake a Principal Component Analysis (PCA), Tables 1 and 2 show the results of the eigenvalues, relative variance contributions, and load matrixes of various main elements (Table 1, Table 2), which formed the basis for extracting the principal component of urbanization. Per capita GDP, as well as the proportion of employees in production and service industries, the proportion of life service employees, the proportion of professional and technical personnel, per capita fiscal income, and the proportion of the immigrated population were all found to be are heavily related to the first principal component. As such, these factors can be described as constituting “economic vitality factors.” In comparison, we found both distance to railroads and relief amplitude to be strongly related to the second principal component, and as such they can be considered to constitute “geographical location factors.” Similarly, the abundance of water resources was found to be strongly related to the third principal component, and thus can be described as comprising the “water resource factor.” Per capita investment in fixed assets was strongly related to the fourth principal component, and can thus be seen as making up “the investment factor.” Finally, the number of welfare house beds per 10,000people was found to be strongly related to the fifth principal component, and can be considered to constitute the “social welfare factor.” We conducted further cluster analysis of the results of the main element analysis, trying to divide the variants of new-type urbanization into eight distinct categories using the SPSS software platform. We also introduced the results of categorization into ArcGIS 10.1 for visual representation purposes (Fig 2). These results could subsequently be used as the basic reference for division and primary basis for setting borders.

**Table 1:** Eigenvalue and relative variance contributions of various main elements.

Main elements	Eigenvalue	contribution rate of variance (%)	accumulative contribution rate (%)
1	4.855	34.682	34.682
2	2.183	15.596	50.277
3	1.332	9.517	59.794
4	1.199	8.562	68.356
5	1.003	7.162	75.518
6	0.719	5.135	80.653
7	0.631	4.506	85.159
8	0.508	3.632	88.791
9	0.345	2.461	91.252
10	0.310	2.212	93.464
11	0.291	2.077	95.541
12	0.226	1.615	97.157
13	0.220	1.574	98.731
14	0.178	1.269	100.000

**Table 2:** Load matrixes of various main elements.

Indicators	Main elements				
	1	2	3	4	5
Gross domestic product (GDP) per capita	0.771	-0.130	-0.083	0.441	-0.056
Fixed assets investment per capita	0.638	0.105	-0.207	0.551	0.069
Percentage of employees in manufacturing industries	0.460	-0.464	0.477	-0.086	-0.033
Percentage of employees in producer service industries	0.790	-0.126	0.221	-0.333	-0.048
Percentage of employees in consumer service industries	0.692	0.382	0.083	-0.328	0.050
Average years of education	0.593	-0.424	-0.372	-0.303	-0.052
Percentage of professional technicians	0.780	0.314	-0.005	-0.317	0.016
Fiscal income per capita	0.814	0.001	0.010	0.447	-0.053
Percentage of migrating population	0.810	0.136	0.223	0.033	-0.184
Number of medical beds per 10 thousand people	0.464	0.354	-0.320	-0.228	0.272
Welfare per 10 thousand people	0.107	-0.216	0.172	0.075	0.932
Distance to railroads	0.012	0.634	-0.349	-0.051	0.083
Relief amplitude	-0.155	0.852	0.117	0.129	-0.012
Water abundance	-0.091	0.426	0.737	0.065	0.014

### Regionalization Plans

**Fig 3:**
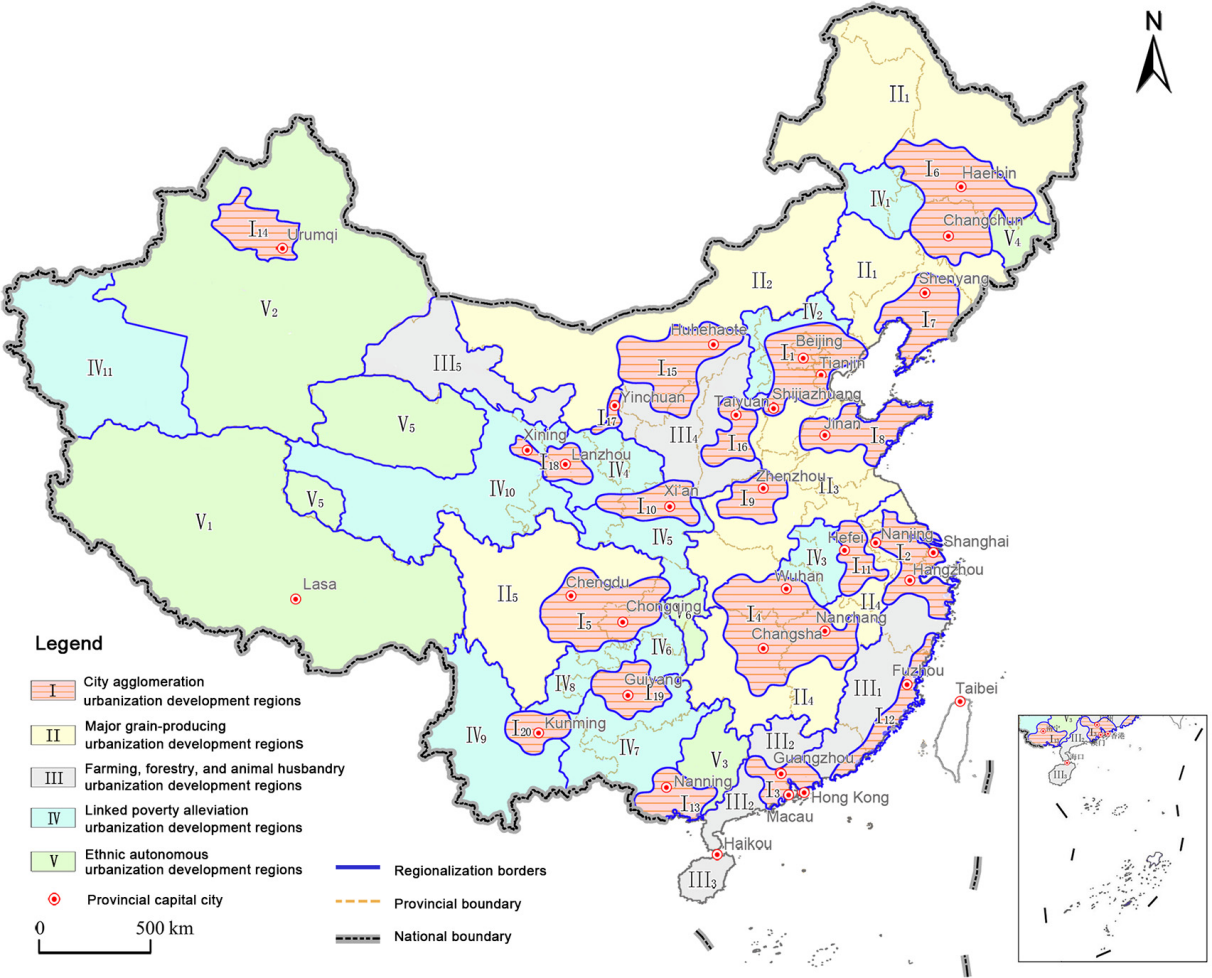
Comprehensive Regionalization Plan of China’s New-Type Urbanization Development.

Using the regionalization method designed through this study, we divided national urbanization regions into city agglomeration urbanization development regions (Ⅰ); major grain-producing urbanization development regions (Ⅱ); farming, forestry, and animal husbandry urbanization development regions (Ⅲ); linked poverty alleviation urbanization development regions (Ⅳ); and ethnic autonomous urbanization development regions (Ⅴ). This division gave a total of 5 types of regions and 47 sub-regions (Fig 3, Table 3). The category “city agglomeration development regions” (Ⅰ) consisted of five national city agglomerations, nine regional city agglomerations, and six local city agglomerations. “Major grain-producing urbanization development regions” (Ⅱ) included Hebei, Inner Mongolia, Liaoning, Jilin, Heilongjiang, Shandong, and Henan (the seven major grain-producing regions in the North), and also Jiangsu, Anhui, Jiangxi, Hubei, Hunan, and Sichuan (the six major grain-producing regions in the South). “Farming, forestry, and animal husbandry urbanization development regions” (Ⅲ) were determined after the major grain-producing areas according to national comprehensive agriculture regionalization and ecological regionalization, and included the five sub-regions of the southeast hills areas, the Nanling areas, the Hainan and South China Sea Islands areas, the Chinese Loess Plateau areas, and the Hexi Corridor areas. The 11 sub-regions which made up the category of “linked poverty alleviation urbanization development regions” (Ⅳ) were determined according to the 11 main battlefields of national poverty alleviation projects identified in the *Outline for Development-Oriented Poverty Reduction for China's Rural Areas (2011–2020)* published by the State Council for implementation in December 2011. The “ethnic autonomous urbanization development regions” (Ⅴ) were determined according to the Distribution of National Ethnic Areas, including six sub-regions of the Tibetan Autonomous Region, the Xinjiang Uygur Autonomous Region, the Guangxi Zhuang Autonomous Region, the Yanbian Korean Autonomous Region, the Hercynian Mongolian Tibetan Autonomous Region, and the Xiangxi Tujia and Miao Autonomous Region.

**Table 3:** Comparison of Detailed List and Feature Statistic Indexes of Regionalization in China’s New-Type Urbanization.

Code	Type	Area (%)	Population (%)	Population density (people per km2)	Urban population (%)	Urbanization(%)	GDP (%)	Economic density (Ten thousand yuan per km2)
**I**	**City agglomeration UDR**	**25.82**	**62.83**	**340**	**78.42**	**45.43**	**80.57**	**1420.50**
I_1_	Beijing-Tianjin-Hebei	1.90	6.30	463	10.11	60.48	9.06	2169.77
I_2_	Yangtze River Delta	1.14	6.33	772	11.28	66.50	16.17	6430.00
I_3_	Pearl River Delta	0.58	2.25	546	4.71	71.83	8.62	6819.93
I_4_	Middle Reaches of Yangtze River	2.94	8.44	402	8.35	36.33	7.32	1135.07
I_5_	Chengdu-Chongqing	2.50	8.07	450	10.32	43.86	5.31	965.17
I_6_	Harbin-Changchun	2.92	3.46	166	4.23	41.84	3.74	583.97
I_7_	Mid-southern Liaoning	1.22	2.77	318	4.11	52.85	4.49	1674.91
I_8_	Shandong Peninsula	1.17	4.68	556	5.31	46.29	7.47	2896.22
I_9_	Central Henan	0.61	3.39	773	3.00	30.29	3.06	2273.18
I_10_	Guanzhong	0.93	2.19	330	2.05	32.02	1.58	773.44
I_11_	Jiang-Huai	0.74	2.27	427	2.73	41.25	2.02	1242.69
I_12_	West Coast of Taiwan Strait	0.87	3.90	625	3.50	39.52	4.10	2144.65
I_13_	Beibu Gulf	0.76	1.69	312	0.91	38.37	0.98	587.25
I_14_	North Tianshan Mountain	0.62	0.31	70	0.70	76.60	0.56	410.48
I_15_	Hu-Bao-E-Yu	3.08	1.11	50	1.25	38.52	2.35	347.61
I_16_	Central Shanxi	0.93	1.48	222	1.73	40.16	1.27	622.67
I_17_	Ningxia Yellow River	0.54	0.37	94	0.50	46.89	0.33	279.51
I_18_	Lanzhou-Xining	0.79	1.04	185	0.92	30.46	0.57	328.48
I_19_	Central Guizhou	0.57	1.23	299	1.36	38.03	0.58	461.00
I_20_	Central Yunnan	1.00	1.54	215	1.35	36.62	0.98	444.59
**II**	**Major grain-producing UDR**	**20.80**	**18.97**	**121**	**10.02**	**30.43**	**13.02**	**284.91**
II_1_	Northeast China Plain	7.30	2.08	40	1.75	35.33	3.91	243.73
II_2_	Inner Mongolia	4.81	0.12	4	0.13	34.61	0.85	80.07
II_3_	Huang-Huai-Hai Plain	3.12	11.35	508	5.1	27.49	4.17	608.22
II_4_	Middle and Lower Reaches of the Yangtze River	2.15	4.41	221	2.32	36.86	3.52	742.83
II_5_	Southwest China	3.42	1.01	41	0.72	31.19	0.59	77.90
**III**	**Farming, forestry, and animal husbandry UDR**	**6.21**	**6.77**	**133**	**4.73**	**27.16**	**4.12**	**298.53**
III_1_	Hilly Region of Southeast China	1.35	1.96	181	1.35	26.31	1.15	387.67
III_2_	Nan Mountains	0.84	1.85	306	1.45	26.86	0.99	533.13
III_3_	Hainan-Nanhai Islands	0.52	0.95	172	0.63	33.20	0.82	715.10
III_4_	Loess Plateau	1.12	1.70	174	1.05	25.84	0.70	283.79
III_5_	Hexi Corridor	2.44	0.32	18	0.25	27.13	0.45	84.71
**IV**	**Linked poverty alleviation UDR**	**18.25**	**8.82**	**67**	**4.04**	**21.91**	**1.13**	**28.18**
IV_1_	Greater Khingan Mountains	0.84	0.27	45	0.19	23.64	0.13	69.12
IV_2_	Yan and Tai-hang Mountains	0.92	0.53	80	0.25	24.89	0.11	53.83
IV_3_	Ta-pieh Mountains	0.66	1.74	370	0.54	22.49	0.09	64.71
IV_4_	Liu-pan Mountains	0.73	0.62	119	0.36	19.82	0.10	59.80
IV_5_	Qin-ba Mountains	1.01	0.82	114	0.32	21.55	0.11	49.44
IV_6_	Wu-ling Mountains	0.39	0.44	155	0.31	24.07	0.10	112.28
IV_7_	Yunnan-Guangxi-Guizhou Stony Desertification	1.91	1.59	116	0.74	20.30	0.05	12.34
IV_8_	Wu-meng Mountains	0.34	0.57	234	0.34	20.47	0.11	148.84
IV_9_	Western Yunnan Border Mountains	2.51	1.53	85	0.66	23.72	0.06	10.76
IV_10_	Tibetan Region of Four Provinces	4.45	0.23	7	0.12	18.59	0.15	14.91
IV_11_	South Xinjiang	4.50	0.50	16	0.21	19.76	0.13	13.31
**V**	**Ethnic autonomous UDR**	**28.92**	**2.61**	**13**	**2.79**	**36.60**	**1.16**	**18.26**
V_1_	The Tibet Autonomous Region	12.52	0.22	3	0.15	22.67	0.16	5.78
V_2_	Xinjiang Uygur Autonomous Region	11.59	0.65	8	0.75	39.24	0.36	14.07
V_3_	Guangxi Zhuang Autonomous Region	0.81	1.31	226	1.26	32.85	0.24	133.45
V_4_	Yanbian	0.45	0.17	52	0.35	70.35	0.18	185.78
V_5_	Haixi Tibet and Mongolian Autonomous Region	3.14	0.04	2	0.07	70.08	0.13	18.95
V_6_	Xiangxi Tujia Autonomous Region	0.41	0.22	73	0.21	33.67	0.09	100.24
**China**		**100.00**	**100.00**	**140**	**100**	**34.61**	**100.00**	**455.25**

Source: Based on China Statistical Yearbook 2013.

Note: **UDR** stands for Urbanization Development Region.

### Features of Various Regions

**Fig 4:**
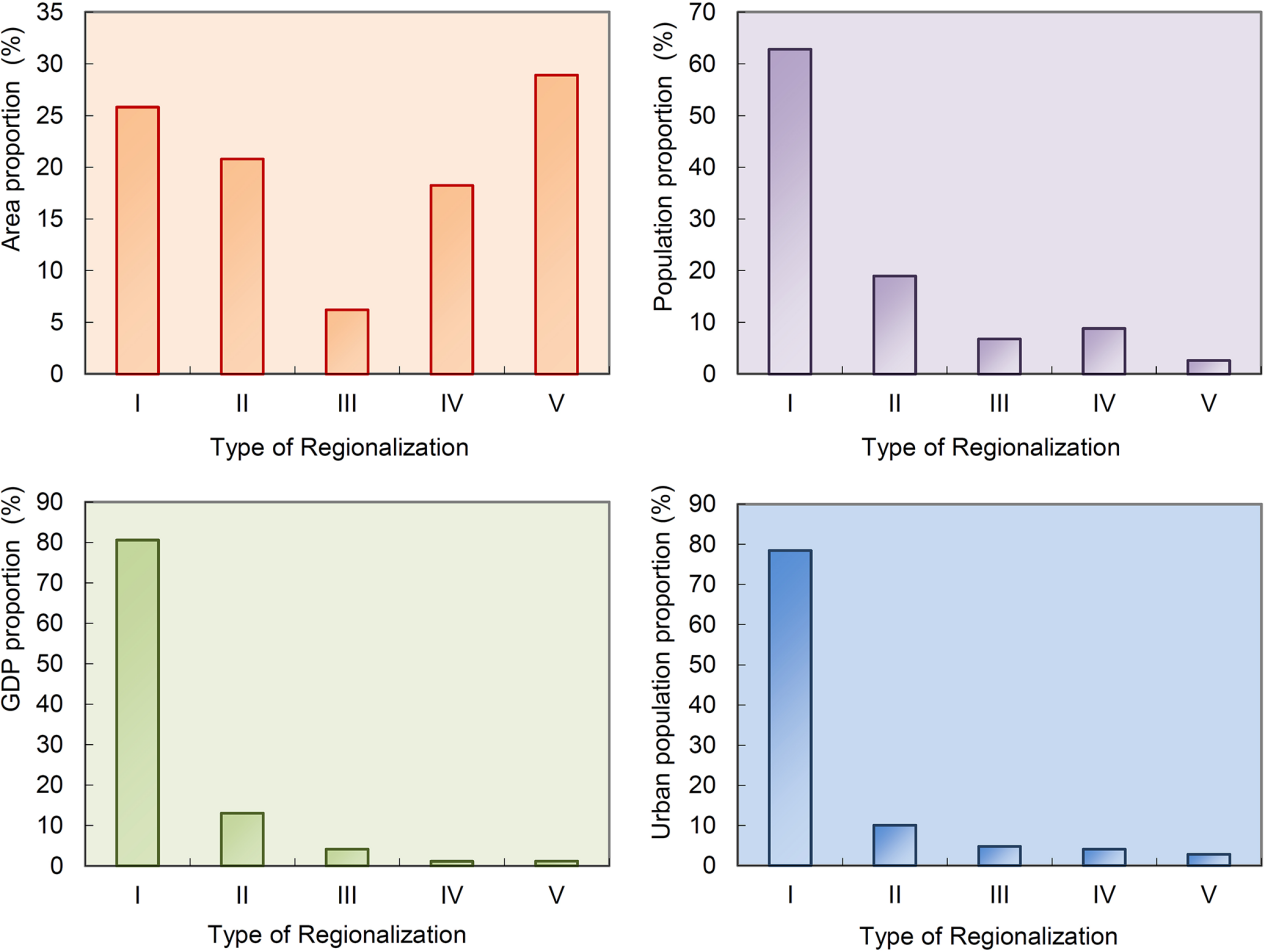
Comparison of Development Status of Various Regions in New-Type Urbanization in China.

**Fig 5:**
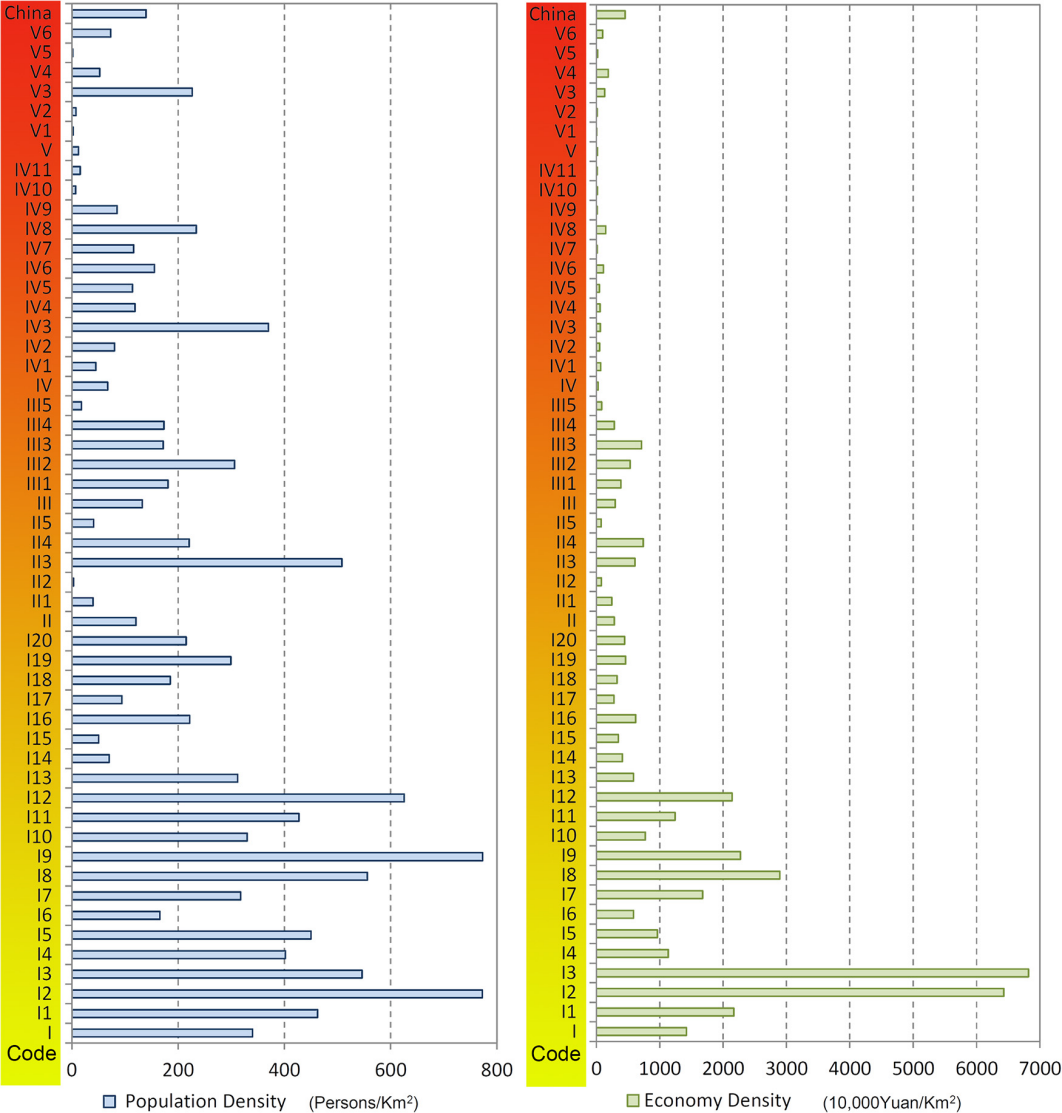
Comparison of Population Density and Economy Density of Various Regions in New-Type Urbanization in China.

Various regions and sub-regions play different roles in national urbanization development (Table 3, Fig 4, Fig 5), and the urbanization of various regions likewise demonstrates distinctive features.

“City agglomeration urbanization development regions” (Ⅰ) are the main regions for national new-type urbanization. The twenty sub-regions proposed through this study all constitute strategic core regions for current and future economic development and new-type urbanization. They also constitute the main supporting regions in relation to the urbanization of agricultural populations. Meanwhile, these regions are also areas with serious environmental pollution, which urgently needs to be curbed. The total area of the this region was found to account for 25.82% of the country’s land area, but in 2012 its total population accounted for a staggering 62.83% of the national population, and its urban population accounted for 78.42% of the country. In the same year, these regions collectively maintained an urbanization level of 45.43%, 10.22 percentage points higher than the national average level; the population density was 339.87 people / sq km, which was 2.43 times of the national average; and the economic density was 14.205 million yuan / sq km, which was 3.12 times of the national average. Among the five types of regions put forward in this paper, these regions have the highest population and economic density, urbanization level and economic aggregation. Accordingly, they are the absolutely primary regions for national new-type urbanization: they will in this sense determine the future of China’s urbanization, and they will shoulder the primary load in achieving China’s urbanization.

**Fig 6:**
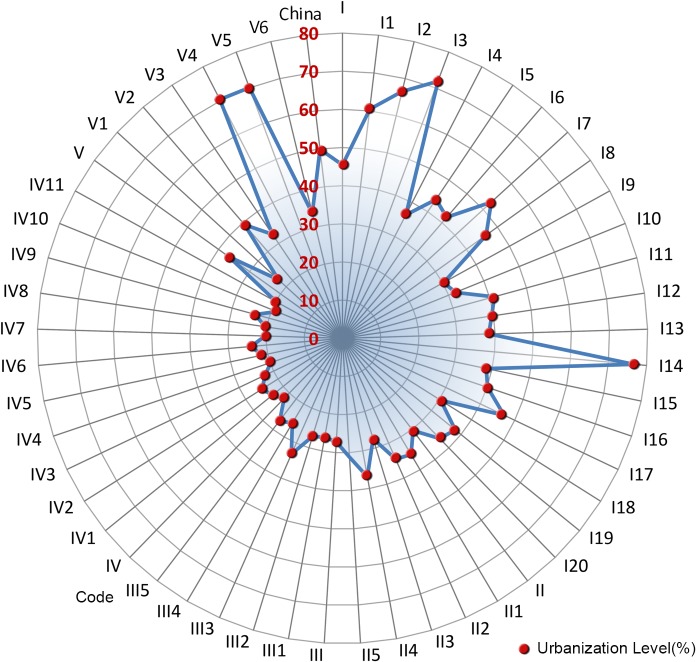
Comparison of Urbanization Level of Various Regions in New-Type Urbanization in China.

“Major grain-producing urbanization development regions” (Ⅱ) were formulated in this research as underdeveloped or undeveloped regions characterized by plains as their primary terrain, agriculture-dominated economies, and farmers as their main population. The grain-planting acreage of these regions accounts for 71.84% of the national grain-planting acreage; in 2012, their grain production was 446.1 million tons, accounting for 75.4% of total national grain production; the rate of increase in grain production in these regions represents approximately 95% of the national grain production increase. Given these characteristics, these regions play a critical role in increasing total national grain production and ensuring national food security. The population density and economic density of these regions is relatively low, and the agricultural population is large. The urbanization level is 30.43%, which is 4.2% lower than the national average level (Fig 6). The economic aggregation of these regions ranks second among the five largest of the region types identified in the study.

Most “farming, forestry, and animal husbandry regions”(Ⅲ) are located in the mountains, hills, and plateau areas. These regions produce the bulk of China’s cash crops, and their ecological systems are relatively weak. Their economic density reached 2.9853 million yuan / sq km in 2012, lower than the national average by a number of 1.5672 million yuan / sq km; the population density was 132.65 people / sq km, higher than the major grain producing areas; and the level of urbanization (27.16%) and per capita income (22,500 yuan / person) were lower than the major grain production areas. On the whole, whilst the population density and economic density of these regions are relatively high, their urbanization level and per capita income are relatively low.

Most “poverty alleviation urbanization development regions” (Ⅳ) are located in the mountainous regions of China, where the living and developmental environment is severe, transportation is inconvenient, and the resident income level is relatively low. The population density of these regions, as they were identified in this study, was 67.48 people / sq km, lower than the national average by a number of 72.2 people / sq km; the economic density reached 281,800 yuan / sq km in 2012, lower than the national average by a number of 4.2707 million yuan / sq km; the per capita GDP was 4200 yuan / person, which was the lowest of all the region types, accounting for just 12.89% of the national average level. On the whole, the population density and economy density of these regions is relatively low, and their urbanization level is the lowest. Their economic development lags, and people’s living standard is, as a result, the lowest.

“Ethnic autonomous urbanization development regions” (Ⅴ) are all located in minority ethnic group areas and remote areas with vast territory, small population, and various ethnic groups. The population density of these regions as they were identified in the study was 18.26 people / sq km, lower than the national average by a number of 127.08people / sq km, and the economic density reached 18,260 yuan / sq km in 2012, 4.37 million yuan / sq km lower than the national average. The urbanization level of these regions was, by contrast, 36.6%, ranking just after the city agglomeration areas. On the whole, their population and economy densities are the lowest, and their economic development lags. However, the urbanization level of these regions is instead higher than the national average level, illustrating the unique characteristics of new-type urbanization in ethnic autonomous regions.

## Discussion

### Difference with Other Regional Categorization

The regional categorization of new-type urbanization must focus, as it has in this study, on the key elements and regional key issues at stake in such urbanization; it is in this way that research might, we believe, best serve the healthy development of this new urbanization model. The results of the approach developed here differ from those obtained in previous studies using other methods. Despite the clear differences, though, some elements and issues do present interactions in relation to other regional categorization systems, and as such it is valuable to relate the present results to those of existing studies. For example, *China’s Comprehensive Agricultural Regionalization* and *China’s Ecological Function Regionalization* were taken as references when defining the farming, forestry, and animal husbandry urbanization development regions. *China’s Comprehensive Agricultural Regionalization* divided China into nine agricultural regions based on agricultural production conditions, characteristics, and major problems. These regions serve for the scientific prediction of the direction of agricultural production, the guidance of agricultural production, and the long-term agriculture development planning. *China’s Ecological Function Regionalization*, which is based on the ecological surveys, divides regional units with different leading ecological functions by analyzing regional ecological characteristics, ecosystem services, and ecological sensitivity. Reference to these allows the direction of characteristic industries and the path of increasing income to be defined without violating the laws of plant growth and damaging the environment, based on the distribution and ecological values of agricultural land, woodland, and grassland. In the 2010 release of the *National Main Functional Area Planning*, urbanization and its development pattern were only parts of the national land development, and the core issues of urbanization were not examined; comparatively, the regional categorization of new-type urbanization pays more attention to human development.

### The Development Focus of Each Region

The five types of regions identified in this study each exhibit different features and problems in relation to new-type urbanization, addressing these aspects at the regional level would facilitate the healthy sustainable development of each region in relation to its urbanization. “City agglomeration urban development regions” (Ⅰ) are here identified as the strategic core areas of new-type urbanization development in China, bearing the primary task of implementing the national strategic objectives of the new-type urbanization plan, via a focus on resolving the problems of urban population agglomeration. In2020, these regions should have an urbanization level of more than 60%. Given that China’s natural population growth rate is very low, new urban population growth essentially relies on agricultural migrants. Since city agglomeration urban development regions are densely concentrated with new urban population, their living, working, housing, and development should be the subject of close attention and be improved through reforms to the household registration system and through service protection measures for migrant workers [40], which will be the (difficult) key to improving the quality of urbanization in such regions. Furthermore, as densely concentrated urban areas, city agglomerations should face up to important issues such as coordinating basic services and infrastructures between cities, coordinating public services and infrastructures between city and rural areas, and cooperation on regional ecological environment management.

“Major grain-producing urbanization development regions” (Ⅱ) face dual pressures in terms of urbanization and food production; they also are the typical site for land-use conflicts during the process of land urbanization [15]. These areas on the one hand bear the responsibility to ensure national food security, which requires a certain amount of arable land and strict farmland protection policy; on the other hand, in order to promote urbanization, they need to secure and guarantee a degree of new land for urban development. Since land and space are limited resources, contradictions between the cultivation of land, urbanization, and industrialization are increasingly evident. Therefore, for these regions, new-type urbanization processes should be complemented by agricultural modernization, the construction of new countryside by the transformation of old homesteads, the remediation of “hollow” villages, the reasonable replacement of urban and rural construction land, the reform of land and fiscal systems [22], and improvements to the efficiency of intensive land uses. These measures would allow for the simultaneous construction of food security barriers and acceleration of new urbanization.

“Farming, forestry, and animal husbandry urbanization development regions” (Ⅲ) present the problem of impetus, which revolves around the question of how to increase incomes. For example, in 2014 the Chinese government issued the *Prohibition for Forest Cutting and Chopping* and adjusted many original forest production bases to ecological function conservation areas, which increased the unemployment of local dwellers and decreased the important local fiscal income, resulting in capital and financial difficulties which can be directly linked to new-type urbanization in such regions. The pastoral areas also have deviation problems in relation to their industrial and employment structures—since the ability of industrialization to act as a driver for urbanization is limited, non-farming employment opportunities are few, which restrict the development of urbanization. Given these conditions, these regions—as they have been identified here—should explore urbanization development patterns such as those focusing on agricultural areas, pastoral areas and forestry areas[24] by adjusting measures to local conditions, in accordance with each region’s different geographical features, topography, and natural-ecological conditions. In addition, large-scale government investment in public works and models focused on people’s involvement model can also improve income and ensure healthy local urbanization.

“Linked poverty alleviation urban development regions” (Ⅳ), as identified in this study, constitute the locus for resolving regional lack of public services, in line with the goal of achieving the equalization of public services. These regions are host to a series of problems, such as wide-ranging poverty, a high degree of poverty, weak infrastructure, an imperfect market system, low levels of economic development, lagging social development, alack of basic public services, a fragile ecological environment, and large gaps between urban and rural areas. In order to achieve the equalization of public services by targeting these regions, it is necessary to first address problems of how much and how many to allocate, how to allocate, where to obtain financial support, over what period of time, what will be considered progress. Besides the poverty alleviation project simplemented by the government, the self-development capacity of these regions can, for instance, be improved through the development of tourism and characteristic industries [41].“Ethnic autonomous urbanization development regions” (Ⅴ) are identified as special regions in relation to new-type urbanization, wherein the purpose of intervention should be national unity and social stability, and measures should be formulated and undertaken in order to explore the integration of ethnic minority cultures and urbanization. These regions, as they are defined here, gather most of China’s ethnic minorities, which give these regions unique cultures and religions, as well as urban constructions, industrial development, social life, and public services, all of which are closely related to local culture. Urbanization of these regions should not only be undertaken in accordance with the overall plan for China’s “new-type urbanization,” but also needs to highlight the particularity of the ethnic cultures present in these regions. Therefore, all available resources should be made available to pursue urbanization that is sensitive to the unique local cultures in these regions, and those cultures should be integrated into new-type urbanization processes.

### Differentiated Policies Adopted by Various Regions

According to their different strategic status and key issues in relation to the national development of “new-type urbanization,” the five major types of regions identified in this study each have their own specific goals, emphases, models, and paths. As such, and with a view to their diversity, future policy work addressing new-type urbanization in these regions should combine the principles of universality and diversity. Public goods, including substantive public goods like production and living infrastructures, basic educational resources, health and medical conditions, safety and environmental settings, public services, and social security should be shared by all people; but in the urbanization process, different policies should be implemented in order to guide local urbanization. In the areas we have identified as “city agglomeration urban development regions,” the key is to promote the reform of the household registration system and solve the problem of urban immigrants; in “major grain-producing urbanization development regions,” the key is to promote land reform and solve the problem of excessive land encroachment; in “farming, forestry, and animal husbandry urbanization development regions,” the key is to promote market reforms and income increases; in “linked poverty alleviation urban development regions,” the key is to promote investment and financial reform and solve the problem of public service allocation; in ethnic autonomous urbanization development regions, the key is to promote the reform of the management system and integrate ethnic cultures into urbanization.

## Conclusions

The new-type urbanization plan recently issued by the Chinese government is the programme of action in relation to China’s urbanization. The plan will affect various aspects of China’s development, including urban construction and development, social development, and economic growth. Based on the position that “problems associated with new-type urbanization must be addressed differently in different regions, and the key issues in relation to new-type urbanization are different in every area,” this study designed a special method to divide China into several new urbanization region types (and thus regions) that are able to serve as a basis for discussion and development of new-type urbanization, and deliver healthy sustainable development in accordance with local conditions.

(1) This paper presents a method for regional categorization related to the treatment of social problems within the humanities, enriching established methodologies related to regionalization, as well as the Chinese comprehensive regionalization system. Regionalization is an important means of recognition and effective management via the categorization of complex territories and areas. Geographical zoning is often used to solve complex regional systems of human-land relationships [32, 33]. The method developed and deployed in this study differs markedly from existing methods. Since the emphasis of new-type urbanization emphasis is rather on problems of a social nature, our method is mainly qualitative, treating quantitative approaches as supplementary (although existing natural zoning and economic divisions were determined via quantitative analysis). Combing the existing regionalization and planning with new methods, this approach reflects the historical origins of the humanities and uses that research tradition to offer better solutions to the problems associated with urbanization regionalization. This method breaks through the limits of past methods in order to create a new notion of regionalization, providing a reference for solving complex regional social problems in the future.

(2) The results fill in a gap in current academic knowledge about the regionalization of China’s urbanization. This study divides the country into 5 types of large regions and 47 small regions, which helps clearly elucidate the urbanization features of different regions and specifically discuss the development strategy, objective, model and approach for each region in relation to conditions of new-type urbanization. The division is based on an understanding of the substance and features of new-type urbanization and stresses the key issues at stake in different regions’ urbanization. Accordingly, the results are also able to reflect the aims of new-type urbanization.

(3) The regional categorization of new-type urbanization is specifically designed to answer to China’s urbanization, in a manner which differs from existing regionalization plans. At present, China has many specific regionalization plans that play important roles in the nation’s social and economic development in certain periods, for instance, the *Main Function Zoning* helps in addressing national land development issues, *China’s Comprehensive Agricultural Regionalization* gives directions on agricultural development issues, and *China’s Ecological Function Regionalization* helps in ecological protection [42]. As a complex process and a model for future development, new-type urbanization interacts with all of the above issues, and as such these plans have been taken as key references in this study. However, new-type urbanization represents the latest important model and process to be addressed in China’s development; as such this research can be expected to deviate from existing plans. We believe that the regionalization categories set out here have the potential to play an important role in China’s healthy and sustainable urbanization.

## Supporting Information

S1 Fig Map of Urban Agglomeration Development Plan of China. (TIF)

S2 Fig Map of National Major Grain-Producing Area Planning of China. (TIF)

S3 Fig Map of National Comprehensive Agricultural Planning of China. (TIF)

S4 Fig Map of Chinese Ecological Areas. (TIF)

S5 Fig Map of National Concentrated and Linked Poverty Alleviation Plan. (TIF)

S6 Fig Map of Distribution of National Ethnic Areas. (TIF)
