# Supplementary figures and images for: A Regional Categorization for “New-Type Urbanization” in China
Source: PLoS One. 2015 Aug 3;10(8):e0134253. doi: 10.1371/journal.pone.0134253 (PMC4523198; doi:10.1371/journal.pone.0134253)

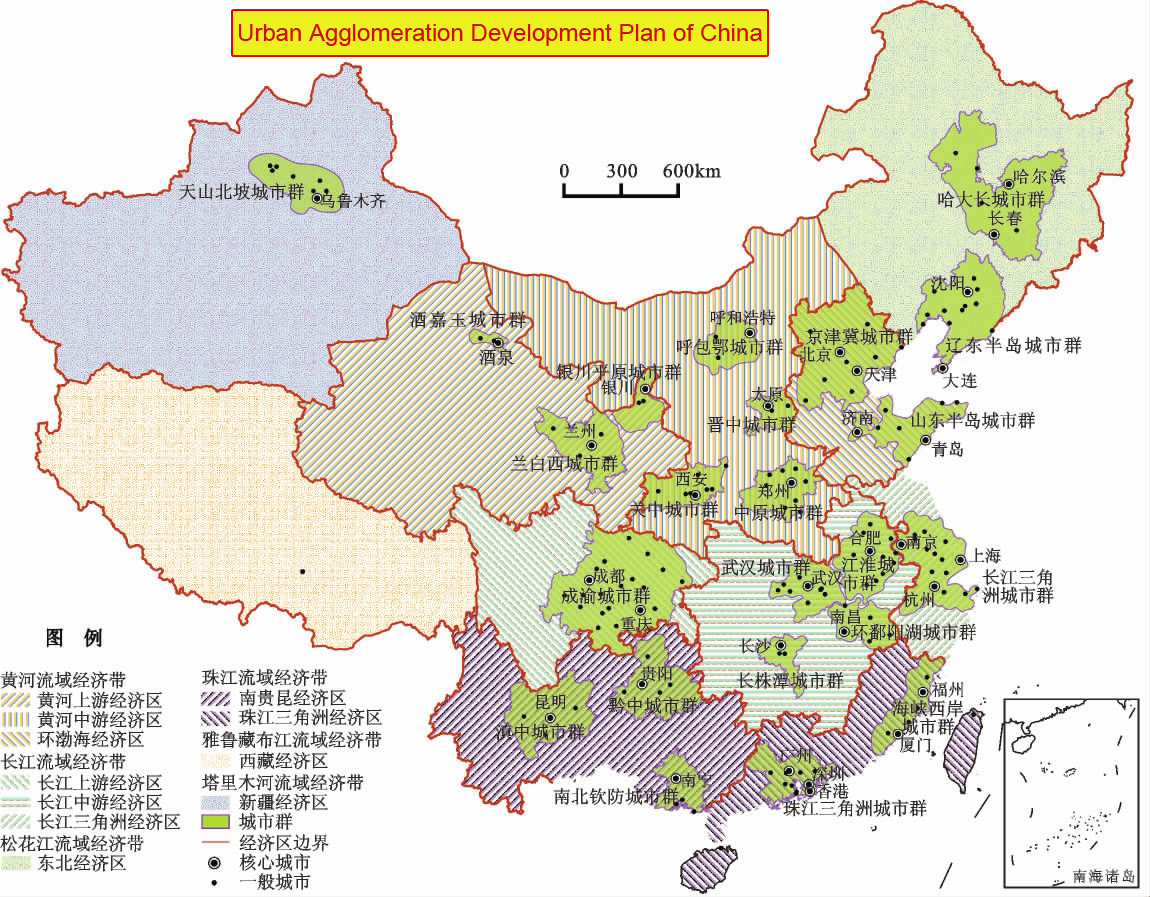

Supplement: S1 Fig — (TIF) [file pone.0134253.s001.tif]

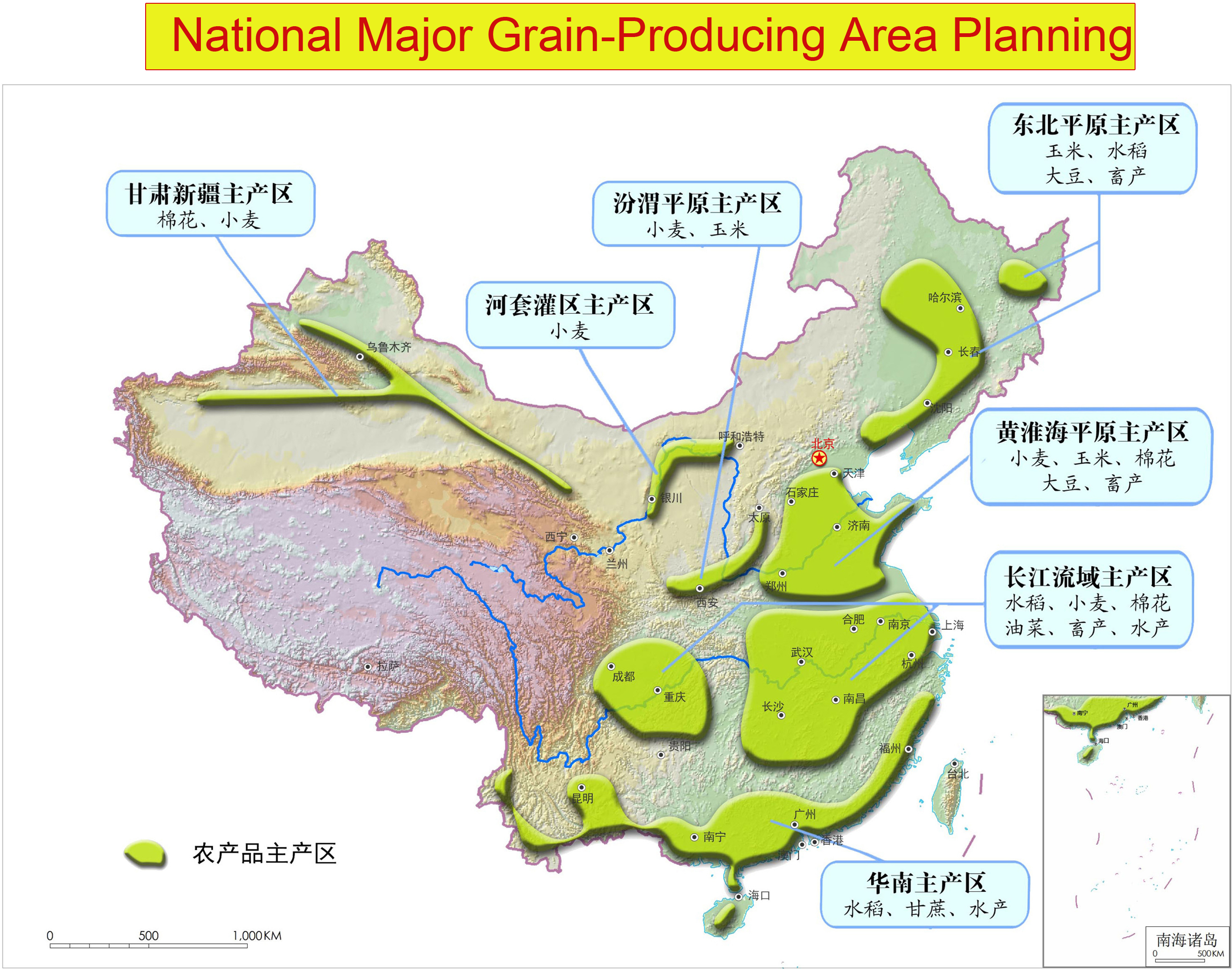

Supplement: S2 Fig — (TIF) [file pone.0134253.s002.tif]

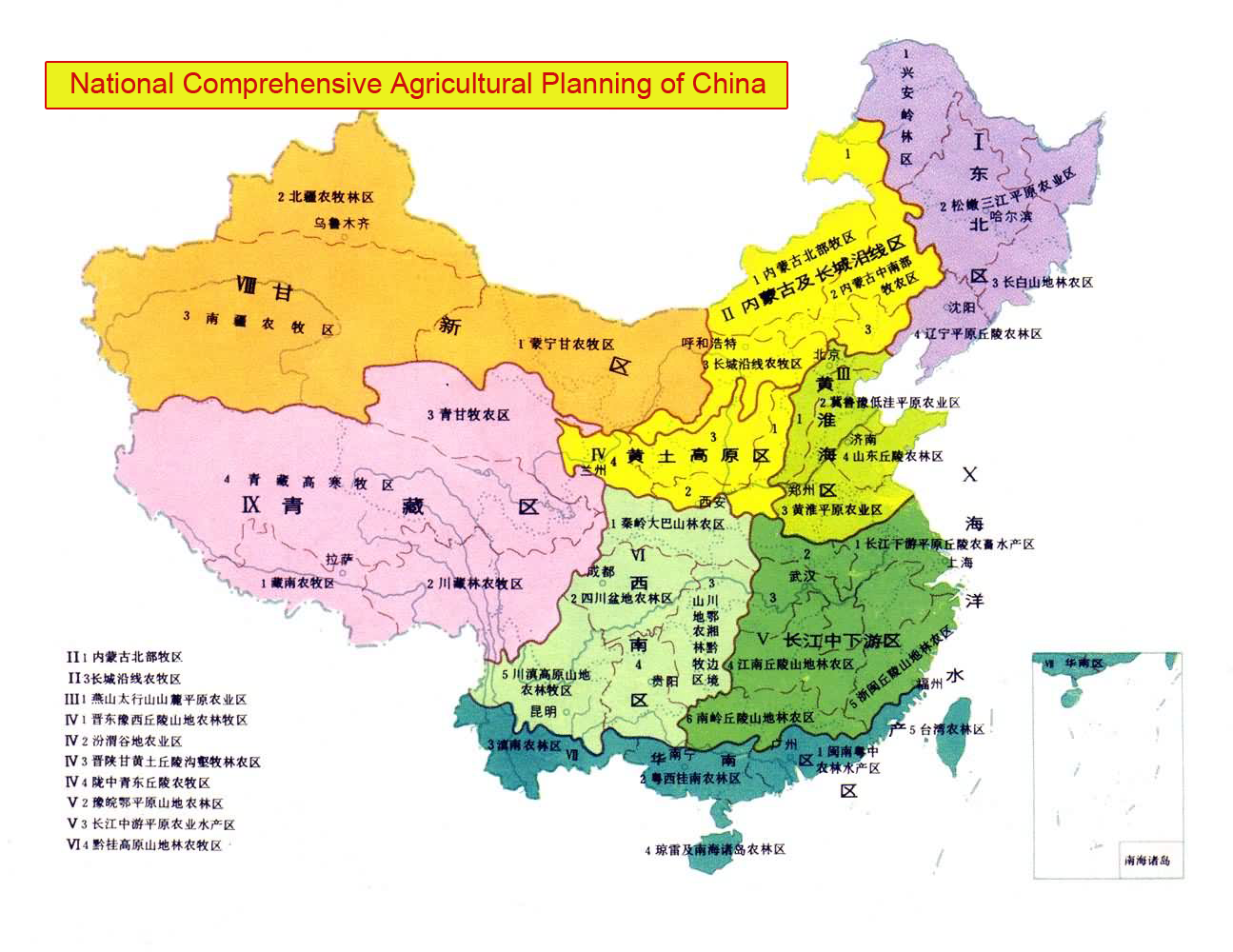

Supplement: S3 Fig — (TIF) [file pone.0134253.s003.tif]

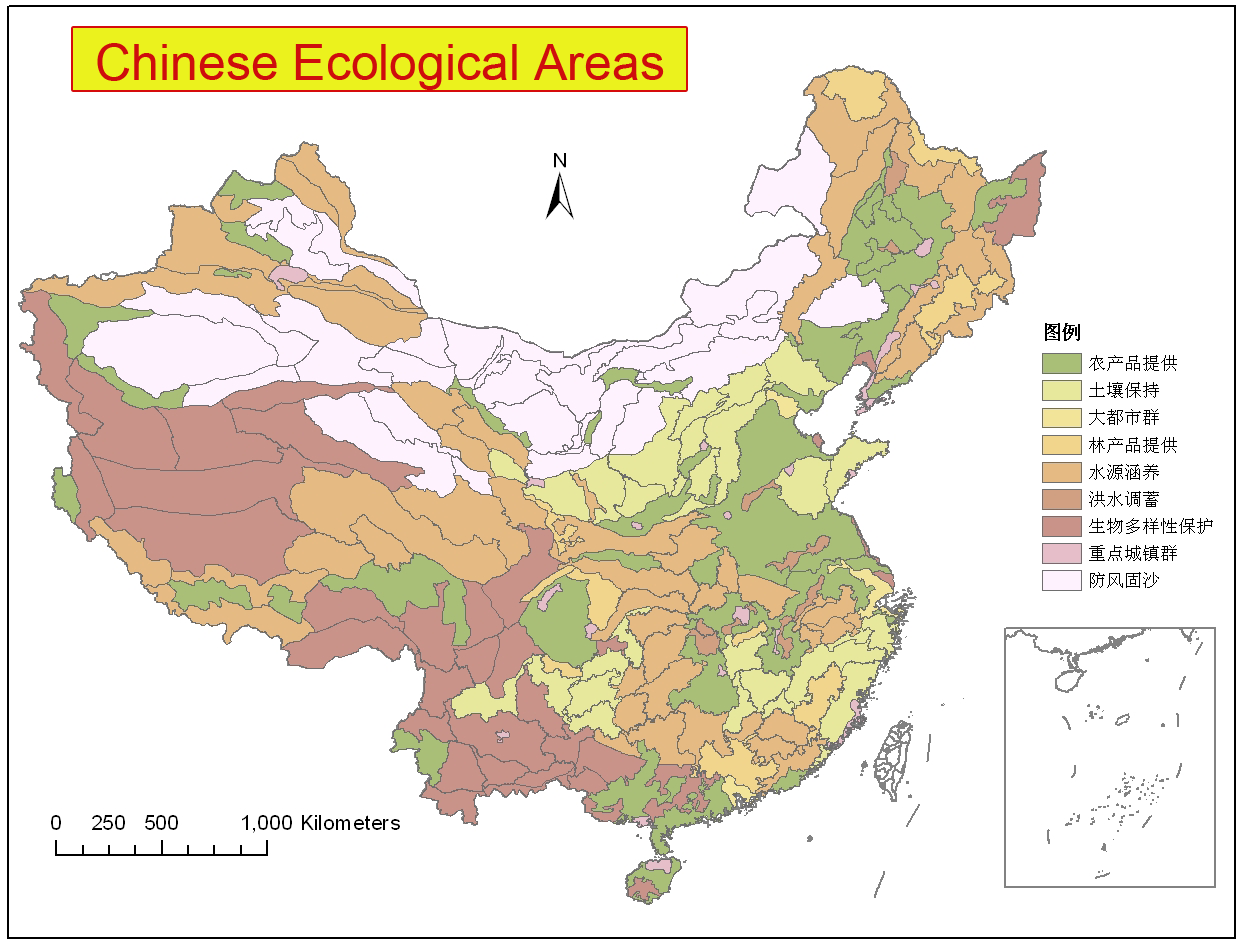

Supplement: S4 Fig — (TIF) [file pone.0134253.s004.tif]

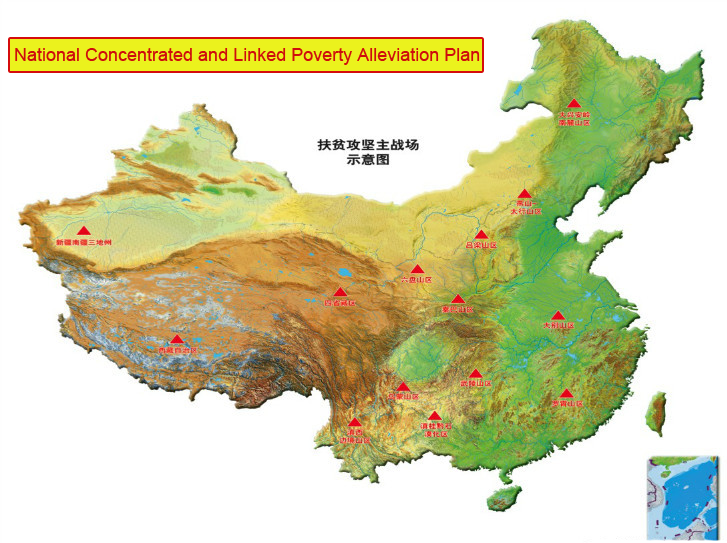

Supplement: S5 Fig — (TIF) [file pone.0134253.s005.tif]

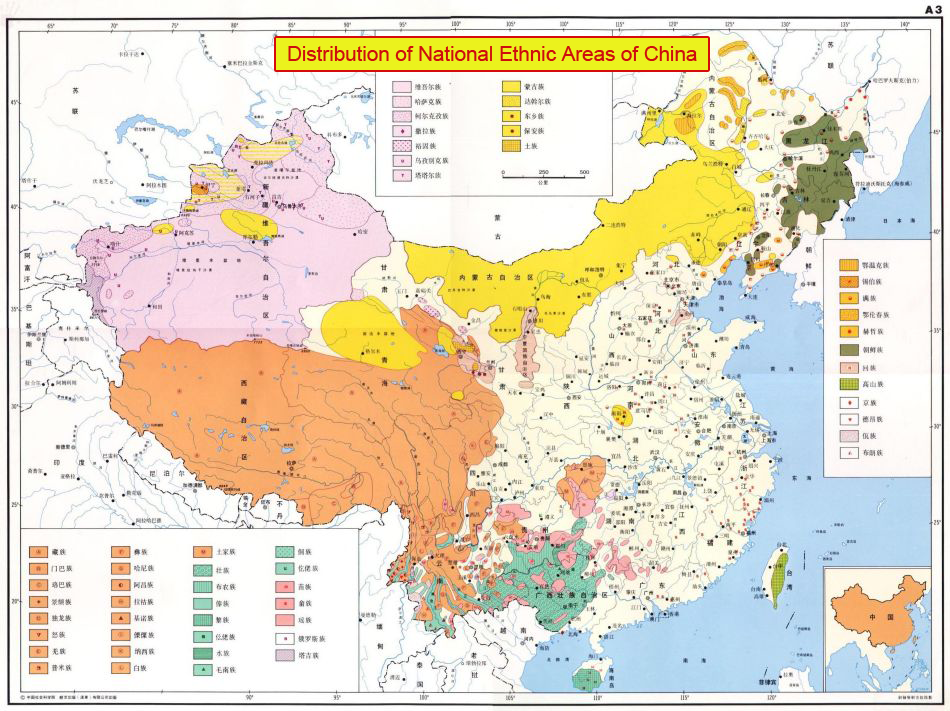

Supplement: S6 Fig — (TIF) [file pone.0134253.s006.tif]
